# Incidence and Antepartum Risk Factors of Severe Postpartum Haemorrhage in Anaemic Pregnant Women in Lagos, Nigeria: A Secondary Cohort Analysis

**DOI:** 10.7759/cureus.54980

**Published:** 2024-02-26

**Authors:** Kehinde S Okunade, Olufemi A Oyedeji, Olusola F Olowoselu, Adebola Adejimi, Iyabo Ademuyiwa, Ayokunle M Olumodeji, Hameed Adelabu, Aloy Ugwu, Muisi Adenekan, Ayodeji A Oluwole

**Affiliations:** 1 Department of Obstetrics and Gynaecology, College of Medicine, University of Lagos, Lagos, NGA; 2 Department of Obstetrics and Gynaecology, Lagos University Teaching Hospital, Lagos, NGA; 3 Department of Haematology and Blood Transfusion, College of Medicine, University of Lagos, Lagos, NGA; 4 Department of Community Health, Lagos University Teaching Hospital, Lagos, NGA; 5 Department of Nursing Science, College of Medicine, University of Lagos, Lagos, NGA

**Keywords:** postpartum bleeding, predict-pph, uterine fibroids, obesity, lagos

## Abstract

Background: The magnitude and risk factors for postpartum haemorrhage (PPH) have been extensively investigated, although little is currently known about the incidence and predictors of severe PPH, specifically among women affected by prenatal anaemia in Nigeria.

Objectives: The study determined the incidence and antepartum risk factors of severe PPH in anaemic pregnant women in five health institutions in Lagos, Southwest Nigeria.

Methods: A secondary analysis was performed using the data of pregnant women with anaemia from the "*Predict-PPH*" study that was conducted between January and June 2023. This study included n=570 pregnant women affected by anaemia who gave birth in five hospitals in the Lagos metropolis of Nigeria. The study outcome was severe PPH, defined as an estimated blood loss of at least 1000 mL within 24 hours of childbirth. A backward stepwise conditional approach in a multivariable logistic regression model was utilised to identify the independent risk factors for severe PPH in anaemic pregnant women.

Results: Of the 570 women with prenatal anaemia enrolled in the primary study, 42 (7.4%) had severe PPH. The identified independent risk factors for severe PPH were maternal obesity (adjusted OR = 3.85, 95% CI = 1.85-8.02), antepartum haemorrhage in index pregnancy (adjusted OR = 2.98, 95% CI = 1.29-6.90), uterine fibroids (adjusted OR = 6.10, 95% CI = 2.39-15.52), delivery gestational age ≥39 weeks (adjusted OR = 2.62, 95% CI = 1.23-5.56), and delivery by caesarean birth (adjusted OR = 16.75, 95% CI = 5.81-48.31).

Conclusion: About one in 13 anaemic pregnant women enrolled in the study developed severe PPH during childbirth. Maternal obesity, antepartum bleeding in the current pregnancy, co-existing uterine fibroids in pregnancy, delivery gestational age beyond 38 weeks, and caesarean birth in the current pregnancy were factors that were significantly associated with severe PPH in anaemic pregnant women. These findings underscore the importance of increased vigilance during both the antenatal and peripartum periods to identify women with these risk factors for the initiation of timely interventions to prevent severe PPH.

## Introduction

Prenatal anaemia is defined as haemoglobin (Hb) concentrations less than 11.0 g/dL in the first trimester and less than 10.5 or 11.0 g/dL in the second or third trimester (depending on the guideline used) [1]. It is ranked as the most prevalent medical disorder during pregnancy and contributes significantly to global maternal and perinatal morbidity and mortality [2]. Recent emerging evidence suggests a link between prenatal anaemia and an increased risk of PPH [3]. Some implicated mechanisms attributed to anaemia include increased blood flow from bleeding vessels due to the increased heart rate and cardiac output and decreased blood viscosity, impaired oxygen transport that induces uterine atony, and impaired haemostasis and coagulation due to reduced circulating red blood cells [4-5].

PPH is the leading cause of maternal mortality worldwide [2]. It is more prevalent in Western and Central Africa and South Asia, where about half of the women of reproductive age are anaemic [6]. According to the Royal College of Obstetricians and Gynaecologists (RCOG) recommendation, major (or severe) PPH is defined as postpartum blood loss exceeding 1000 mL in the 24 hours after childbirth [7]. Although the magnitude and risk factors for PPH have been extensively investigated, little is currently known about the incidence and predictors of severe PPH, specifically among women affected by prenatal anaemia in Nigeria, the country with one of the highest burdens of maternal mortality worldwide [8].

Given the disturbing magnitude of prenatal anaemia and the public health impact of major PPH, further investigation of the magnitude and the potential factors influencing this important adverse pregnancy outcome may be necessary to guide maternal health policy decisions. We, therefore, determined the incidence and antepartum risk factors of severe PPH in anaemic pregnant women in five health institutions in the metropolitan city of Lagos, Southwest Nigeria.

## Materials and methods

Study design, settings, and population

We conducted a secondary analysis of the data of anaemic pregnant women enrolled in the “Predict-PPH” study [5], a recent prospective cohort study conducted in the prenatal clinics of a mix of secondary and tertiary health institutions in Lagos, Nigeria, from January to June 2023. Further study descriptions are available in the original publication [5]. We included and analysed the data of n=570 women aged 18 to 49 years with prenatal anaemia enrolled at 28 to 36 weeks gestational age in the main study [5]. Excluded from the main study were women with a major medical condition such as sickle cell anaemia, significant renal and hepatic impairment, coagulation disorders, and antepartum foetal demise. Further excluded from the datasets of this current study were women who were lost to follow-up in the course of this study.

Extracted variables of interest

Variables that were relevant to our analyses in the original dataset were extracted. These were the participant's age (in years), gestational age at enrollment (in weeks), the number of previous childbirths, mode of conception, pregnancy type, pregestational or first-trimester body mass index (BMI; calculated as maternal weight (using the actual pre-gestational or first-trimester measurement) in kilograms divided by the square of height in meters), marital, educational, and employment status, antepartum bleeding in the current pregnancy (APH), ultrasound-detected uterine fibroids, Hb concentration at enrollment (in g/dL), and the site and type of the enrollment facility. Hb is estimated using the HemoCue® B-Haemoglobin system (HemoCue®, Ängelholm, Sweden), and prenatal anaemia was defined as an Hb concentration below 11 g/dL as per the WHO recommendation [9]. Eligible participants were classified according to their Hb concentration into mild anaemia (10 to <11 g/dL), moderate anaemia (7 to <10 g/dL), and severe anaemia (<7 g/dL) categories [9]. Obesity was defined as a BMI at or exceeding 30 kg/m² [10]. The main study outcome was RCOG-defined severe PPH, which is postpartum blood loss exceeding 1000 mL in 24 hours after childbirth [7], measured directly using a calibrated blood collection V-drape receptacle. The V-drape is a calibrated under-buttock drape that is folded out into a large, sterile surface for delivery. A fluid pouch at the bottom of the sterile area holds more than 2500 mL of fluid and is marked at 50 mL intervals, thus allowing for precise measurement of postpartum blood loss. The collection pouch includes a flexible plastic filter to trap non-liquid materials and indicates when 500 mL of postpartum blood loss has been collected [5].

Statistical analysis

The outcomes of interest in our study were the incidence and significant risk factors for severe PPH. We adopted SPSS Statistics version 28.0 (IBM Corp. Released 2021. IBM SPSS Statistics for Windows, Version 28.0. Armonk, NY: IBM Corp) for our statistical analyses. We employed the Kolmogorov-Smirnov test with Lilliefors’ significance correction to assess the normality of continuous variables. Subsequently, we calculated descriptive statistics for all relevant maternal, clinical, and obstetric characteristics. Categorical variables were reported using frequencies and percentages, while continuous variables were represented by mean and standard deviation for normally distributed data or median and interquartile range for skewed distributions. Using binary logistic regression models, univariable and multivariable analyses were performed to identify potential risk factors for severe PPH. Age and other factors associated with an increased risk of severe PPH (p<0.20) in the univariable analyses were included in the pool of factors for the final regression model using the backward conditional approach. Akaike's information criterion (AIC) was consistently computed, and the final model steps with the lowest AIC were chosen as the best-fit models. The strength of associations was reported using odds ratios (ORs) at 95% confidence intervals (CIs). Associations in the final models were considered significant if p<0.05.

Ethical approval

Ethical approvals for the original study (Predict-PPH) were received from the Health Research Ethics Committee of the Lagos University Teaching Hospital (ADM/DSCST/HREC/APP/5443) and Lagos State University Teaching Hospital (LREC/06/10/2000). The study adhered to ethical standards outlined in the World Medical Association Declaration of Helsinki. Before enrollment, all participants in the study provided written informed consent, and a strict commitment to preserving the privacy and confidentiality of participant information was upheld throughout and following the completion of the study.

## Results

**Figure 1:**
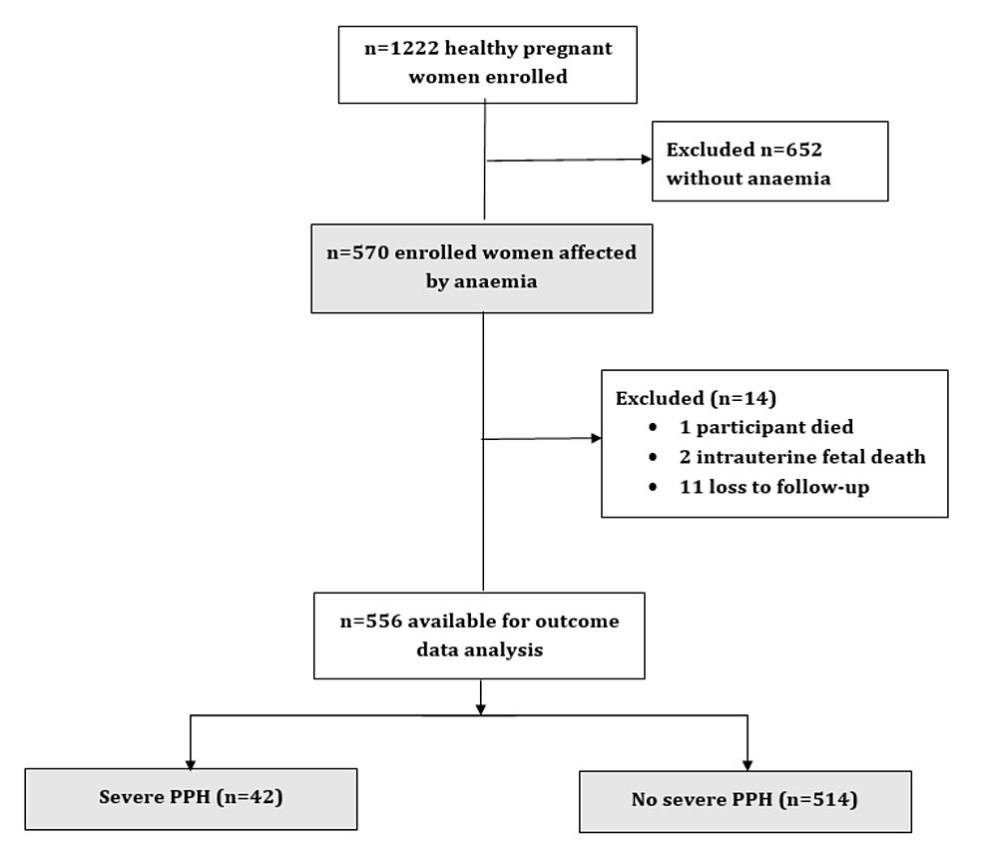
Flowchart of the study participants PPH: postpartum haemorrhage

Of the n=1222 women enrolled at baseline in the main study [5], 570 (46.6%) had Hb concentrations below 11 g/dL. Of these n=570, one died before delivery, two experienced intrauterine foetal demise, and 11 were lost to follow-up. Out of these n=570 anaemic women, 556 (97.5%) had complete clinical data available for analysis after completion of follow-up. The incidence of severe PPH was 7.4% (42/570) (Figure 1).

Following the multivariable analyses of clinical and obstetric characteristics of study participants, the identified independent risk factors of severe PPH in the study included obesity (adjusted OR = 3.85, 95% CI = 1.85-8.02), antepartum haemorrhage in index pregnancy (adjusted OR = 2.98, 95% CI = 1.29-6.90), uterine fibroids (adjusted OR = 6.10, 95% CI = 2.39-15.52), delivery gestational age ≥39 weeks (adjusted OR = 2.62, 95% CI = 1.23-5.56), and delivery by caesarean birth (adjusted OR = 16.75, 95% CI = 5.81-48.31) (Table 1).

**Table 1:** Univariable and multivariable analyses of potential antepartum risk factors for severe PPH in anaemic pregnant women RCOG defined PPH as blood loss of at least 1000 mL in the first 24 hours of childbirth. The multivariable model controls for level of enrolment facility, enrolment gestational age, participant’s age, multiparity, obesity, mode of conception, type of pregnancy, any antepartum bleeding, uterine fibroids, previous hypertensive disorder in pregnancy, hypertensive disorder in current pregnancy, previous caesarean delivery, previous PPH, and delivery gestational age. RCOG: Royal College of Obstetricians and Gynaecologists; BMI: body mass index; CI: confidence interval; NA: not applicable; OR: adjusted odds ratio; PPH: postpartum haemorrhage

Factors	Number of women with severe PPH	Crude	Adjusted
		p-value	OR (95% CI)
Level of enrolment facility			
Secondary	30/371 (8.1%)	0.501	NA
Tertiary	12/185 (6.5%)		
Enrolment gestational age			
≥32 weeks	26/272 (9.6%)	0.08	2.07 (0.98-4.39)
<32 weeks	16/284 (5.6%)		1 (reference)
Participants age			
≥35 years	13/128 (10.2%)	0.204	0.84 (0.35-1.99)
<35 years	29/428 (6.8%)		1 (reference)
Previous childbirths			
≥2	18/167 (10.8%)	0.059	2.05 (0.96-4.37)
<2	24/389 (6.2%)		1 (reference)
Obesity			
Yes (BMI ≥30 kg/m^2^)	23/142 (16.2%)	<0.001	3.85 (1.85-8.02)
No (BMI <30 kg/m^2^)	19/414 (4.6%)		1 (reference)
Mode of conception			
Assisted	2/9 (22.2%)	0.093	1.12 (0.14-8.67)
Spontaneous	40/547 (7.3%)		1 (reference)
Type of pregnancy			
Multiple	1/13 (7.7%)	0.985	NA
Singleton	41/543 (7.6%)		
Severity of prenatal anaemia			
Moderate-to-severe anaemia	16/176 (9.1%)	0.351	NA
Mild anaemia	26/380 (6.8%)		
Antepartum bleeding			
Yes	13/55 (23.6%)	<0.001	2.98 (1.29-6.90)
No	29/501 (5.8%)		1 (reference)
Uterine fibroids			
Yes	11/64 (17.2%)	<0.002	6.10 (2.39-15.52)
No	31/492 (6.3%)		1 (reference)
Previous hypertensive disorder in pregnancy			
Yes	1/17 (5.9%)	0.791	NA
No	41/539 (7.6%)		
Current hypertensive disorder in pregnancy			
Yes	2/29 (6.9%)	0.891	NA
No	40/527 (7.6%)		
Previous caesarean delivery			
Yes	22/119 (18.5%)	<0.001	1.55 (0.64-3.75)
No	20/437 (4.6%)		1 (reference)
Previous PPH			
Yes	0/9 (0.0%)	0.387	NA
No	42/547 (7.7%)		
Delivery gestational age			
≥39 weeks	26/290 (9.0%)	0.188	2.62 (1.23-5.56)
<39 weeks	16/266 (6.0%)		1 (reference)
Mode of delivery			
Caesarean birth	37/218 (17.0%)	<0.001	16.75 (5.81-48.31)
Vaginal birth	5/338 (1.5%)		1 (reference)

At enrolment, 389 (68.2%) of the participants had mild anaemia, 175 (30.7%) had moderate anaemia, and six (1.1%) had severe anaemia. The baseline mean participants’ age was 30.3 ± 5.2 years, and the gestational age was 31.5 ± 2.5 weeks. Table 2 shows the baseline characteristics of the enrolled cohorts.

**Table 2:** Baseline characteristics of study participants (n=570) BMI: body mass index; FMCEb: Federal Medical Center Ebute-Meta; IQR: interquartile range; LASUTH: Lagos State University Teaching Hospital; LIMH: Lagos Island Maternity Hospital; LUTH: Lagos University Teaching Hospital; 68-NARHY: 68 Nigerian Army Reference Hospital Yaba; SD: standard deviation; Hb: haemoglobin

Characteristics	Number (%)
Mean age (± SD) in years	30.3 ± 5.2
Mean gestation age at enrollment (± SD) in weeks	31.5 ± 2.5
Mean BMI (± SD) in kg/m^2 ^	27.0 ± 5.6
Median Hb concentration (IQR) in g/dL	10.0 (9.6–10.6)
Enrolment facility	
LUTH	67 (11.8)
LASUTH	64 (11.2)
LIMH	271 (47.5)
FMCEb	62 (10.9)
68-NARHY	106 (18.6)
Level of enrolment facility	
Secondary	377 (66.1)
Tertiary	193 (33.9)
Previous childbirths	
≥2	399 (70.0)
<2	171 (30.0)
Marital status	
Unmarried	4 (0.7)
Married	566 (99.3)
Educational status	
Less than tertiary education	177 (31.1)
At least tertiary education	393 (68.9)
Employment status	
Unemployed	46 (8.1)
Employed	524 (91.9)
Mode of conception	
Assisted	9 (1.6)
Spontaneous	561 (98.4)
Type of pregnancy	
Multiple	13 (2.3)
Singleton	557 (97.7)
Presence of uterine fibroids	
Yes	64 (11.2)
No	506 (88.8)
Antepartum bleeding in the index pregnancy	
Yes	55 (9.6)
No	515 (90.4)
Previous hypertensive disorder in pregnancy	
Yes	17 (3.0)
No	553 (97.0)
Current hypertensive disorder in pregnancy	
Yes	29 (5.1)
No	541 (94.9)
Previous caesarean delivery	
Yes	120 (21.1)
No	450 (78.9)
Previous PPH	
Yes	9 (1.6)
No	561 (98.4)

Following the multivariable analyses of clinical and obstetric characteristics of study participants, the identified independent risk factors of severe PPH in the study included obesity (adjusted OR = 3.85, 95% CI = 1.85-8.02), antepartum haemorrhage in index pregnancy (adjusted OR = 2.98, 95% CI = 1.29-6.90), uterine fibroids (adjusted OR = 6.10, 95% CI = 2.39-15.52), delivery gestational age ≥39 weeks (adjusted OR = 2.62, 95% CI = 1.23-5.56), and delivery by caesarean birth (adjusted OR = 16.75, 95% CI = 5.81-48.31) (Table 1).

## Discussion

This secondary cohort analysis of the Predict-PPH study [5] determined the incidence of severe PPH and its associated antepartum risk factors in anaemic pregnant women in health institutions in the metropolitan city of Lagos, Nigeria, a country with one of the highest burdens of maternal mortality worldwide [8]. We recorded that about one in 13 anaemic pregnant women enrolled in the study developed severe PPH during childbirth. In addition, the identified independent risk factors for sPPH were obesity, APH, co-existing uterine fibroids in pregnancy, delivery gestational age at or beyond 39 weeks, and caesarean birth in the current pregnancy.

The incidence of severe PPH in anaemic pregnant women (7.3%) reported in our study is much higher than the previously reported modest rates of 1.2% in Uganda [11] and 1.4% in Australia [12] among the general population of pregnant women. However, this figure is only slightly higher than the reported 4.5% in a nationwide study conducted in the United Kingdom [13], 5.3% by Liu et al. in China [14], and 6.1% by Zewdu et al. in South Central Ethiopia among anaemic pregnant women [15]. The observed disparities in the findings of our study and some of these previous studies can be attributed to several potential factors. First, unlike some of these previous studies that were conducted among the general pregnant women population, our study focused exclusively on women affected by anaemia in pregnancy, which has been linked extensively with an elevated risk of PPH [16]. Secondly, the incidence variations may also be due to differences in diagnostic criteria, medical interventions, and healthcare systems in these research settings [17]. For instance, the lack of availability of essential treatments such as heat-stable carbetocin, tranexamic acid, fresh frozen plasma, and platelet concentrates in our clinical setting could contribute to the high incidence of severe PPH. Additionally, our study was conducted in referral facilities, which are mostly patronised by women with high-risk pregnancies.

Our study recorded caesarean birth in the current pregnancy, co-existing uterine fibroids, maternal obesity, APH, and delivery gestational age at or beyond 39 weeks as the antepartum predictors of severe PPH. Compared to vaginal birth, the risk of severe PPH is 16.8 times higher with caesarean delivery. This corroborated the findings by Liu et al. in China [14] and Davey et al. in Australia [12], who reported a similar link between caesarean births and the risk of severe PPH. This is mostly because women undergoing caesarean delivery are likely to experience more blood loss as the standard physiological mechanism involved in uterine contraction and retraction is usually disturbed during a lower-segment caesarean section [18]. Secondly, caesarean sections are associated with an increased tendency for more accurate measurement of blood loss [15], usually due to intensive monitoring by a combination of experts, including anaesthetists and obstetricians. Uterine fibroids are the most prevalent benign growths in women of reproductive age, with a higher frequency among individuals of African descent [19]. The risk of severe PPH in pregnant women with uterine fibroids in this study is greater than the 20% higher risk reported in women with uterine fibroids in a large Chinese study conducted by Zhao et al. in 2017 [20]. The link between fibroids and PPH risk is attributed to the significantly increased risk of adverse pregnancy events associated with uterine fibroids, such as abnormal placentation (placenta previa and abruption) and caesarean section, and the risk of uterine atony due to overdistension of uterine smooth muscle and impaired contractility in the postpartum period [21].

Although the finding of a higher risk of severe PPH in obese mothers observed in our study is not consistent with that reported by Butwick et al. in 2017 [22], It, however, corroborated the findings from a Japanese population-wide study conducted by Enomoto et al. in 2016 [23]. The potential mechanisms for this positive link include the impaired uterine response to control bleeding that occurs due to the disruption of hormonal balance, including insulin resistance, seen in obese women [5]. Secondly, the larger uterine size and volume and more adipose tissue that are associated with obesity make it difficult for the uterus to contract effectively after childbirth. Also, the increased likelihood of requiring operative interventions such as caesarean sections during childbirth in obese mothers is often associated with a higher risk of PPH compared to vaginal deliveries [11]. Similar to previous studies [12,17], women who have APH are at a higher risk of developing severe PPH. This may be due to the link between certain causes of APH, such as placenta previa, abruption, and accreta spectrum, which may also be associated with an increased risk of PPH [12]. For example, a compromised placenta might not function optimally during pregnancy, potentially leading to bleeding during pregnancy, childbirth, and the postpartum period.

Similarly, women with APH are likely to be considered for caesarean delivery, which has a strong link to severe PPH. Our finding of a higher risk of severe PPH in women at or beyond 39 weeks' gestational age further confirmed the notion that gestational age is an underappreciated risk factor for PPH [24] and further corroborated the report by Fukami et al. in Japan [25]. However, a similar association was not observed in the study conducted by Ononge et al. in Uganda [11]. The possible link between advanced gestational age at or beyond 39 weeks could be attributed to the reduced contractility in the myometrium of women who experience post-term pregnancies [26] and the increased risk of labour induction and augmentation, which has been linked to uterine atony and PPH [27], in most women at this gestational age.

The major strength of this study is that it is the first cohort study, to our knowledge, that examined the risk factors for severe PPH, specifically among women with prenatal anaemia in sub-Saharan Africa. However, the study has a few limitations. The study findings are only generalizable to clinical settings in the urban settings of Lagos, as the participating pregnant women were enrolled in hospitals within the Lagos metropolis, thus excluding a major proportion of women who mostly deliver at home in slums and suburban settings of Lagos. Secondly, there are possible confounding variables that were not accounted for in the original study that may influence the observed associations in our secondary analysis. In addition, although we analysed a large sample size of anaemic pregnant women enrolled in the Predict-PPH study, our study may not be sufficiently powered to detect all the possible risk factors for severe PPH in the study settings. We, therefore, advocate for carefully designed and adequately powered future prospective studies that would employ a more comprehensive sampling strategy to include women from a wider range of clinical and community settings, as well as those residing in slums and suburban areas, to allow a trajectory-tracking of prenatal anaemia and assess the development of severe PPH and the temporal relationship with its potential risk factors.

## Conclusions

The study revealed that one in 13 women affected by prenatal anaemia experienced severe PPH, with associated risk factors including maternal obesity, APH, co-existing uterine fibroids, delivery beyond 38 weeks gestation, and caesarean birth. These findings underscore the need for vigilance during pregnancy to identify anaemic pregnant women with these risk factors and for the initiation of timely interventions to prevent the occurrence of severe PPH.
